# Acute Tubercular Infection of the Ileo-Colic Glands Simulating Appendicitis

**Published:** 1915-10

**Authors:** 


**Acute Tubercular Infection of the Ileo-Colic Glands Simulating Appendicitis.** Homer Gage, Worcester, Boston M. and S. Jour., August 26, reports a case and alludes to a number of other cases of involvement of mesenteric glands, with or without the simulation of appendicitis. He quotes Hess as having found the bacilli to be the bovine type in 62 per cent. of 71 cases of mesenteric gland tuberculosis but Hess also reports 3 cases of human type, as does Theobald Smith.

